# Permanganate of Potassium in Diphtheria

**Published:** 1891-08

**Authors:** 


PERMANGANATE OF POTASSIUM IN DIPHTHERIA.—Dr. Netzetzky says that his twenty-two years' practice convinced him that the best treatment of faucial diphtheria consists in an energetic use of permanganate of potassium. The drug should be administered in the shape of paintings and gargle. The following strong solution should be employed:

℞ Potassii Permanganatis, ʒ ij.

Aquæ destillatæ, ℥ j.

M. Sig.: To paint the affected surface every three hours.

For gargling, which is to be repeated as often, a teaspoonful of the same solution should be mixed with a tumblerful of boiled water.

In those cases in which the child is unable to gargle, the following mixture sheuld be given internally:

℞ Solutionis hydrogenis superoxydati, two per cent. ℥ij.
Glycerine, ʒ ij.

M. Sig.: A teaspoonful every three hours.

—*Medical Record.*

